# Anti-Proliferative Activities and Apoptosis Induction by Triterpenes Derived from *Eriobotrya japonica* in Human Leukemia Cell Lines

**DOI:** 10.3390/ijms14024106

**Published:** 2013-02-18

**Authors:** Takuhiro Uto, Ayana Sakamoto, Nguyen Huu Tung, Tsukasa Fujiki, Kenji Kishihara, Shigeru Oiso, Hiroko Kariyazono, Osamu Morinaga, Yukihiro Shoyama

**Affiliations:** 1 Department of Pharmacognosy, Faculty of Pharmaceutical Sciences, Nagasaki International University, 2825-7 Huis Ten Bosch, Sasebo, Nagasaki 859-3298, Japan; E-Mails: uto@niu.ac.jp (T.U.); mabo12141030@gmail.com (A.S.); tunginpc@gmail.com (N.H.T.); morinaga@niu.ac.jp (O.M.); 2 Department of Immunology, Faculty of Pharmaceutical Sciences, Nagasaki International University, 2825-7 Huis Ten Bosch, Sasebo, Nagasaki 859-3298, Japan; E-Mails: fujiki@niu.ac.jp (T.F.); kishihar@niu.ac.jp (K.K.); 3 Department of Pharmaceutical Health Care Sciences, Faculty of Pharmaceutical Sciences, Nagasaki International University, 2825-7 Huis Ten Bosch, Sasebo, Nagasaki 859-3298, Japan; E-Mails: shige@niu.ac.jp (S.O.); karihiro@niu.ac.jp (H.K.)

**Keywords:** corosolic acid, *Eriobotrya japonica*, leukemia cells, apoptosis, anti-proliferation, caspase

## Abstract

*Eriobotrya japonica* leaf is a traditional herbal medicine that contains numerous triterpenes, which have various pharmacological properties. In this study, we investigated the anti-proliferative activity of four triterpenes derived from *E. japonica*, including corosolic acid (CA), ursolic acid (UA), maslinic acid (MA) and oleanolic acid (OA), in human leukemia cell lines. CA showed the strongest anti-proliferative activity in all of the leukemia cell lines tested, but not in normal human skin fibroblast cell lines. To determine the mechanism underlying the anti-proliferative effect of CA, we examined the effect of CA on molecular events known as apoptosis induction. CA induced chromatin condensation, DNA fragmentation, sub-G_1_ phase DNA, activation of caspase-3, -8 and -9 and the cleavage of PARP in HL-60. CA also activated Bid and Bax, leading to the loss of mitochondrial membrane potential (Δψ_m_) and cytochrome c release into the cytosol, whereas Bcl-2 and Bcl-xL were unaffected by CA. These results suggest that CA has an anti-proliferative effect on leukemia cells via the induction of apoptosis mediated by mitochondrial dysfunction and caspase activation. CA may be a potential chemotherapeutic agent for the treatment of human leukemia.

## 1. Introduction

*Eriobotrya japonica* Lindl., also known as loquat, is a fruit tree in the Rosaceae family. Its leaves are listed in the Japanese Pharmacopeia and are used widely as traditional herbal medicine for the treatment of chronic bronchitis and coughs in Japan and other Asian countries [1]. Previously, we found that a crude extract from the leaves strongly inhibited the production of prostaglandin E_2_ (PGE_2_) and nitric oxide (NO) in lipopolysaccharide (LPS)-treated macrophages [2]. In addition, the extract of *E. japonica* leaf was reported to have several anti-cancer effects. *E. japonica* extract showed strong cytotoxicity in cervical cancer (HeLa), lung cancer (A549) and ER-negative breast cancer (MDA-MB-231) cell lines [3]. Kim *et al.* have reported that *E. japonica* extract inhibits the adhesion, migration and invasion of human breast cancer [4]. Moreover, the cell migration and invasion of B16F10 melanoma cells were down-regulated by the extract of *E. japonica* leaves [5].

Phytochemical investigations of *E. japonica* leaves led to the isolation of various triterpenes, some of which were found to possess several pharmacological properties, including anti-inflammatory, anti-tumor, antioxidative and anti-diabetes effects [6–9]. The triterpenes-rich fraction and several isolated triterpenes from *E. japonica* leaves showed the inhibitory effect on 12-*O*-tetradecanoylphorbol-13-acetate (TPA)-induced inflammation and Epstein-Barr virus early antigen (EBV-EA) activation induced by TPA in mice [10]. In addition, the triterpenes from *E. japonica* inhibited inflammatory cytokines/mediators on human lung epithelial cells (A-549) [11] and a rat model of chronic bronchitis [12]. A recent study reported that triterpenes from *E. japonica* exerted anti-fibrosis effects in a rat model of bleomycin-induced pulmonary fibrosis [13]. However, the anti-proliferative activities against cancer cells by triterpenes contained in *E. japonica* leaves are not fully understood.

Apoptosis is a highly regulated process that involves the activation of a series of molecular events leading to cell death. Apoptosis can be initiated by two principal apoptosis pathways, *i.e.*, the death receptor (extrinsic) pathway and the mitochondrial (intrinsic) pathway [14]. The mitochondrial pathway is mainly regulated by Bcl-2 family proteins, which are divided into the pro-apoptotic proteins (e.g., Bax and Bid) and anti-apoptotic proteins (e.g., Bcl-2 and Bcl-xL) [15].

**Figure 1:**
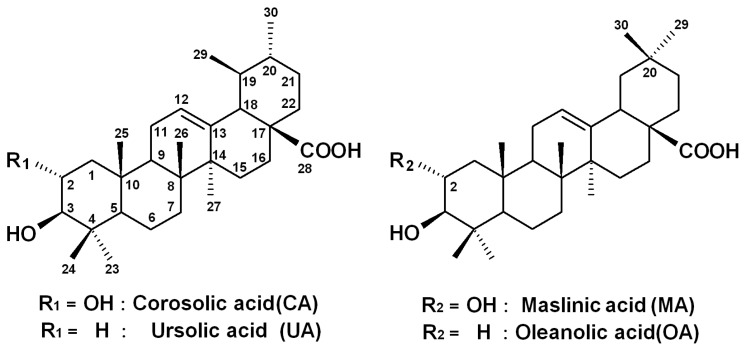
Chemical structures of corosolic acid (CA) and ursolic acid (UA), maslinic acid (MA) and oleanolic acid (OA).

It was reported that the major triterpenes from *E. japonica* leaves are ursane types, such as corosolic acid (CA) and ursolic acid (UA), and oleanane types, such as maslinic acid (MA) and oleanolic acid (OA) (Figure 1) [16,17]. These major triterpenes show anti-proliferative activities against gastric cancer cells (NCI-N87), colorectal cancer (HCT15), cervical cancer (HeLa), glioblastoma (U291, U373 and T98G) and colon cancer (HT29) cell lines [18–23]. As part of the ongoing study, our current screening of medicinal plants found promising antiproliferative effects of a methanol extract from *E. japonica* leaves on several human cancer cell lines. In our preliminary survey, we found that the extract of *E. japonica* leaves suppresses the cell proliferation of leukemia cell lines. In the present study, we investigated the anti-proliferative effects of the major triterpenes from *E. japonica* leaves, *i.e.*, CA, UA, MA and OA, on four human leukemia cell lines. Our data defined the structure-activity correlations of these triterpenes. Furthermore, we investigated the molecular mechanism of CA-induced apoptosis using HL-60 cell lines.

## 2. Results and Discussion

### 2.1. Quality Control of *E. japonica* Leaves

**Figure 2:**
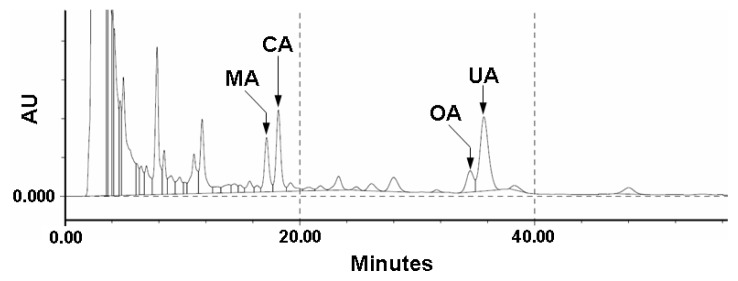
HPLC profile of four triterpenes derived from a CHCl_3_ extract of *E. japonica* leaves.

Because the triterpenes from *E. japonica* leaves might be expected to have potent bioactivities *in vivo* and *in vitro*, the quality control of these triterpenes is necessary for the constant and evident bioactivities. In our ongoing studies on the quality control of natural products, we have been investigating the preparation of monoclonal antibodies against pharmacologically active compounds and their application for quality and/or quantity analysis by using unique methods, like Eastern blotting, a high-sensitive on-membrane quantitative analysis [24–27]. Since *E. japonica* leaves contain various kinds of triterpenes, we performed a fingerprinting of triterpenes contained in *E. japonica* leaves by HPLC to confirm the importance of quality control of *E. japonica*. As shown in Figure 2, the HPLC fingerprint of the CHCl_3_ extract of *E. japonica* leaves showed the profile of the triterpene constituents, where four compounds were highlighted relatively as major components, *i.e.*, CA, UA, MA and OA. The retention time of the ursan-type skeleton (CA and UA) is longer than that of the oleanane group (MA and OA). The retention time of dihydroxyl group in the A-ring (MA and CA) are shorter than that of the monohydroxyl group (OA and UA). Therefore, the quality control of *E. japonica* leaves for the constant bioactive evidence might be needed to confirm the quantitative determination of CA and UA.

### 2.2. Effects of the Four Triterpenes from *E. japonica* Leaves on Cell Proliferation in Human Leukemia and Normal Skin Fibroblast Cell Lines and Their Structure-Activity Correlation

**Figure 3:**
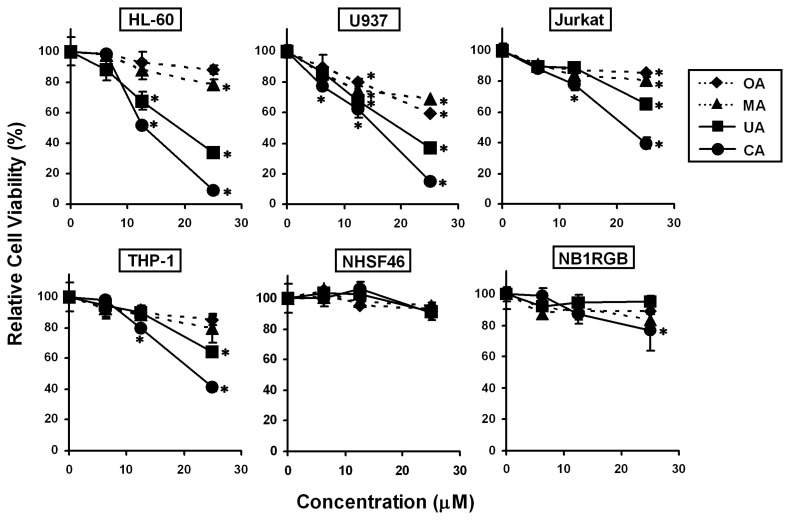
Effects of the four triterpenes on cell proliferation in human leukemia and normal skin fibroblast cell lines. Cells were treated with CA, UA, MA and OA at various concentrations for 24 h, and the cell viability was determined using the MTT assay. The data represent the mean ± S.D. for three individual experiments. ^*^p < 0.05 compared with the control group.

To investigate the effects of CA, UA, MA and OA on cell proliferation in leukemia cell lines (HL-60, U937, Jurkat and THP-1) and normal skin fibroblast cell lines (NHSF46 and NB1RGB), we treated the cells using 6.25, 12.5 and 25 μM of each triterpene for 24 h, followed by the MTT assay (Figure 3). CA and UA significantly suppressed cell growth in all leukemia cell lines, whereas MA and OA had weaker effects than CA and UA. The inhibitory potency against leukemia cell lines followed the order: CA > UA > MA = OA. However, none of the triterpenes inhibited cell proliferation in NGSF46 and NB1RGB remarkably. Thus, we suggest the following structure–activity correlations: (1) the ursane-type skeleton (CA and UA) has a greater suppressive potency than the oleanane-type skeleton (MA and OA); (2) the C_2_–C_3_*trans*-dihydroxyl group in the A-ring is important when comparing CA and UA; and (3) the C_19_–C_20_*trans*-dimethyl group in the E-ring is important when comparing CA and MA. The results indicated that CA was the most potent anti-proliferative triterpene against all of the leukemia cell lines tested, whereas it had low cytotoxicity in normal skin fibroblasts. Among four leukemia cell lines, HL-60 and U937 were more sensitive than Jurkat and THP-1 against the four triterpenes. In future studies, we will investigate the detailed mechanisms underlying the sensitivity difference among the four leukemia cell lines.

### 2.3. Effect of CA on Apoptosis Induction

**Figure 4:**
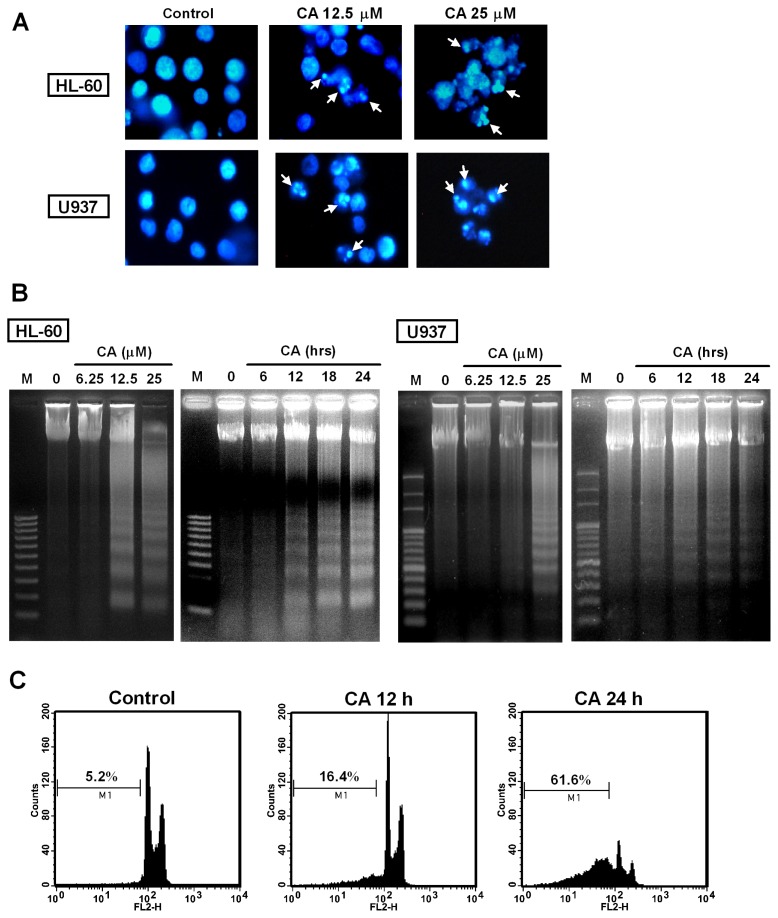
Effect of CA on apoptosis induction. (**A**) Induction of chromatin condensation by CA. HL-60 and U937 cells were treated with the indicated concentration of CA for 24 h and stained with Hoechst 33258. The nuclear morphology was observed by fluorescent microscopy (magnification 400×); (**B**) Induction of DNA fragmentation by CA. HL-60 and U937 cells were treated with CA at various concentrations for 24 h or at 12.5 μM for the times indicated, and the DNA fragmentation was analyzed by agarose gel electrophoresis. M is the 100-bp DNA marker; (**C**) Increase of the sub-G1 phase cells by CA. HL-60 cells were treated with 12.5 μM CA for the times indicated and analyzed by flow cytometry after staining with propidium iodide (PI). The data shown are representative of three independent experiments with similar results.

We investigated whether the CA-induced anti-proliferative activity against leukemia cells was related to apoptosis induction by analyzing the characteristics of apoptosis, including nuclear morphological changes and DNA fragmentation in HL-60 and U937 cells. Both cell lines were treated with CA at 12.5 and 25 μM for 24 h, and the nuclear morphology of the cells was observed using Hoechst 33258 staining. As shown in Figure 4A, the control cells exhibited normal nuclear morphology, whereas the cells treated with CA displayed chromatin condensation. Furthermore, DNA fragmentation was examined based on classical DNA laddering using agarose gel electrophoresis. Figure 4B shows that the CA treatment led to the appearance of the DNA ladder in a dose- and time-dependent manner in both lines. In a parallel experiment, we also analyzed the hypodiploid DNA content (sub-G1 phase) using flow cytometry after the cellular DNA had been stained with propidium iodide (PI). CA treatment of the cells increased in the percentage of cells in the sub-G1 phase from 5.2% (control) to 16.4% (12 h) and 61.6% (24 h), gradually (Figure 4C). Overall, these results clearly indicate that CA exerted its anti-proliferative effect via the induction of apoptotic cell death.

### 2.4. Involvement of the Caspase Cascade in CA-Induced Apoptosis

**Figure 5:**
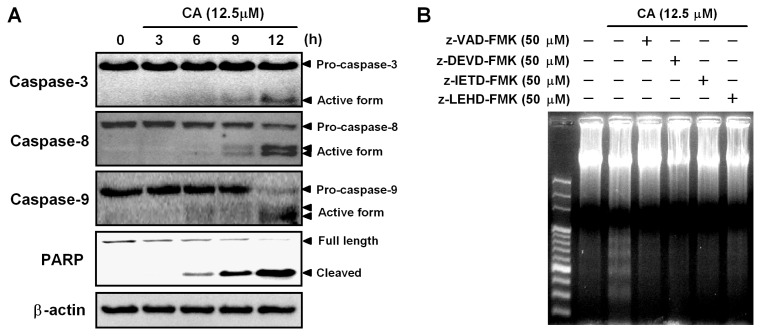
Involvement of the caspase cascade in CA-induced apoptosis. (**A**) Effect of CA on the activation of caspase-3, -8, -9 and PARP cleavage. HL-60 cells were treated with 12.5 μM CA for the times indicated. The cells were lysed, and the caspase-3, -8, -9, PARP and β-actin protein levels were determined by Western blotting. β-actin was used as the loading control; (**B**) The effect of caspase inhibitors on CA-induced DNA fragmentation. After pretreatment with 50 μM caspase inhibitors for 1 h, HL-60 cells were treated with 12.5 μM CA for 24 h. DNA fragmentation was analyzed by agarose gel electrophoresis. The data shown are representative of three independent experiments with similar results.

Caspase-3 is a critical effector caspase and initiates apoptotic damage. Activation of caspase-3 requires the activation of initiator caspases, such as caspase-8 and -9 [28]. To investigate the involvement of the caspase cascade during CA-induced apoptosis, we examined the activation of caspases in CA-treated HL-60 cells by Western blotting. As shown in Figure 5A, CA induced time-dependent activations of caspase-3, -8 and -9. Furthermore, PARP cleavage occurred in response to CA treatment. To confirm the involvement of caspases in CA-induced apoptosis, the cells were treated with CA in the absence or presence of caspase inhibitors, and then, the DNA fragmentation was analyzed. As shown in Figure 5B, CA-induced DNA fragmentation was abolished by pretreatment with z-VAD-FMK (broad caspase inhibitor), z-DEVF-FMK (caspase-3 inhibitor), z-IETD-FMK (caspase-8 inhibitor) and z-LEHD-FMK (caspase-9 inhibitor). These results indicate that CA-induced apoptosis involves a caspase-dependent pathway in HL-60 cells.

### 2.5. Effect of CA on Mitochondrial Dysfunction

**Figure 6:**
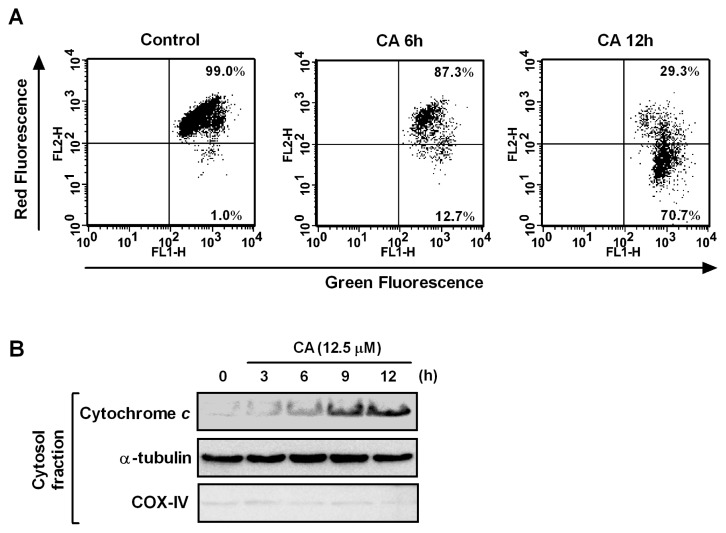
Effect of CA on mitochondrial dysfunction and Bcl-2 family proteins. (**A**) Effect of CA on the loss of Δψ_m_. HL-60 cells were treated with 12.5 μM CA for the times indicated and analyzed by flow cytometry after staining with JC-1; (**B**) Cytochrome c release into the cytosol after treatment with CA. HL-60 cells were treated with 12.5 μM CA for the times indicated, and the cytosolic fraction was analyzed to detect cytochrome c by Western blotting. α-Tubulin and COX-IV were used as cytosolic and mitochondrial fraction controls, respectively. The data shown are representative of three independent experiments with similar results.

Mitochondria play an essential role in apoptosis induction by a variety of death stimuli. Mitochondrial changes include the loss of the mitochondrial membrane potential (Δψ_m_) and cytochrome c release from the mitochondria to the cytosol, which subsequently leads to caspase-9-dependent activation of caspase-3 [29]. Thus, we examined the effect of CA on the Δψ_m_ in HL-60 cells. The cells were treated with CA for 6 h and 12 h, stained with JC-1, which is a sensitive mitochondrial membrane potential probe, and analyzed using flow cytometry. JC-1 can selectively enter mitochondria, depending on the membrane potential, and the JC-1 molecule spontaneously forms J-aggregates that produce intense red fluorescence. As shown in Figure 6A, the CA treatment resulted in a time-dependent decrease in the percentage of red fluorescent cells from 1.0% in the control to 12.7% and 70.7% after treatments of 6 h and 12 h, respectively. Cytochrome c is normally located in the intermembrane space of mitochondria, and loss of the Δψ_m_ causes the release of cytochrome c from mitochondria into the cytosol [30]. Western blotting showed that cytochrome c gradually accumulated in the cytosol in a time-dependent manner in response to CA treatment (Figure 6B). These results indicate that mitochondrial dysfunction is involved in CA-induced apoptosis in HL-60 cells.

### 2.6. Effect of CA on Bcl-2 Family Proteins

**Figure 7:**
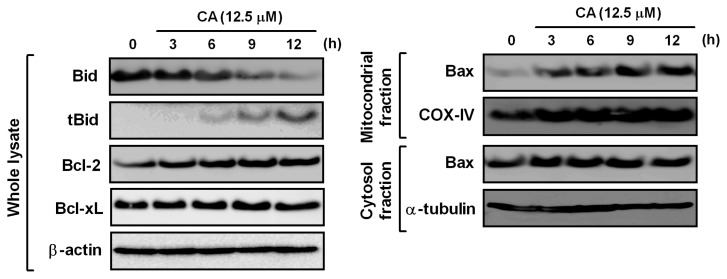
Effect of CA on Bcl-2 family proteins. HL-60 cells were treated with 12.5 μM CA for the times indicated. Whole cell lysates and mitochondrial and cytosolic fractions were prepared, and the levels of each protein were determined by Western blotting. β-actin, COX-IV and α-tubulin were used as the loading controls for the whole cell lysate, mitochondrial fraction and cytosolic fraction, respectively. The data shown are representative of three independent experiments with similar results.

Mitochondrial-mediated apoptosis regulates a balance between pro-apoptotic (e.g., Bax and Bid) and anti-apoptotic (e.g., Bcl-2 and Bcl-xL) proteins [15]. Thus, we analyzed the levels of the Bcl-2 family proteins in CA-treated HL-60 cells. Bid is a pro-apoptotic member, which is cleaved by caspase-8 to its active form, truncated Bid (tBid). As shown in Figure 7, the CA treatment cleaved Bid protein to tBid in a time-dependent manner. By contrast, the anti-apoptotic proteins, Bcl-2 and Bcl-xL, were unaffected by CA. Bid activation triggers the translocation of cytosolic Bax into mitochondria, which is followed by the loss of Δψ_m_ [31]. Thus, we examined the protein level of Bax in the mitochondrial and cytosolic fractions. Our data clearly showed that CA promotes the translocation of Bax into mitochondria. Taken together, our results suggest that CA induces the activation of the pro-apoptotic Bid and Bax, which leads to the loss of Δψ_m_ and the release of cytochrome c from mitochondria into the cytosol.

## 3. Experimental Section

### 3.1. Materials

CA and MA were purchased from Tokiwa Phytochemical (Tokyo, Japan) and Cayman Chemical (Ann Arbor, MI, USA), respectively. UA and OA were obtained from Wako Pure Chemical Industries (Osaka, Japan). *E. japonica* leaves were purchased from Uchida Wakan**-**yaku (Tokyo, Japan). Antibodies against caspase-3 (catalog number 9662), -8 (catalog number 9746), -9 (catalog number 9502), poly(ADP-ribose) polymerase (PARP), Bcl-2 family proteins, β-actin, COX-IV and α-tubulin were obtained from Cell Signaling Technology (Beverly, MA, USA). Fetal bovine serum (FBS) was supplied by GIBCO (Gaithersburg, MD, USA). Caspase inhibitors were purchased from Calbiochem (San Diego, CA, USA). All other chemicals were obtained from Wako Pure Chemical Industries.

### 3.2. HPLC Fingerprints of Triterpenes in the Crude Extract from *E. japonica* Leaves

The standard triterpenes, *i.e.*, CA, UA, MA and OA, were dissolved in MeOH at a concentration of 2.0 mg/mL and stored at 4 °C until use. *E. japonica* leaves were extracted with MeOH. The MeOH extract was suspended in water and then partitioned with CHCl_3_. The CHCl_3_ fraction (30.0 mg) was dissolved in MeOH (1.0 mL) and filtered with a 0.45-μm syringe filter. HPLC analysis was performed using a TOSOH 8020 (Tokyo, Japan) equipped with a TSKgel ODS-100V (5 μm, 250 × 4.6 mm) and an UV-8020 detector (Tosoh, Tokyo, Japan). Separation was conducted using a mobile phase of methanol and 0.15% acetic acid aqueous solution (85:15, *v*/*v*). The solvent flow rate was kept constant at 1.0 mL/min at ambient temperature throughout the analysis.

### 3.3. Purification and Isolation of Triterpenes by Preparative HPLC

The major triterpenes from the CHCl_3_ fraction of *E. japonica* leaves were separated by HPLC, as described above, and were identified by the comparison with each standard compound individually. The purity of all triterpenes (>98%) was confirmed by high-performance liquid chromatography.

### 3.4. Cell Culture and Treatment

HL-60 (human promyelocytic leukemia cells), U937 (human promonocytic leukemia cells), Jurkat (human acute T-cell leukemia cells), THP-1 (human monocytic leukemia cells), NHSF46 (normal human skin fibroblast cells) and NB1RGB (normal human skin fibroblast cells) were obtained from the RIKEN BioResource Center Cell Bank. HL-60, U937, Jurkat and THP-1 were maintained in RPMI1640 medium. NHSF46 and NB1RGB were grown in α-MEM medium. All cell cultures were supplemented with 10% FBS, 1% penicillin-streptomycin and incubated at 37 °C with 5% CO_2_ in fully humidified conditions. For the cell treatments, CA, UA, MA, OA and caspase inhibitors were dissolved in DMSO and stored at −20 °C until use. The DMSO concentrations in the cell culture medium did not exceed 0.2% (*v*/*v*), and the controls were always treated with the same amount of DMSO as that used in the corresponding experiments.

### 3.5. Determination of Cell Viability

Cell viability was determined using a 3-(4,5-dimethylthiazol-2-yl)-2,5-diphenyltetrazolium bromide (MTT) assay. Four leukemia cells (1 × 10^4^ cells/well) and fibroblast cells (0.4 × 10^4^ cells/well) were cultured in 96-well plates and treated with various concentrations of each triterpene for 24 h. At the end of the treatment, MTT solution was added to each well, and the cells were incubated for another 4 h. The precipitated MTT-formazan was dissolved in 0.04 N HCl-isopropanol and the amount of formazan was measured at 595 nm using a microplate reader (Immuno Mini NJ-2300, Nihon InterMed, Tokyo, Japan). Cell viability was expressed as a percentage relative to the control culture.

### 3.6. Nuclear Staining with Hoechst 33258

HL-60 and U937 (1 × 10^6^ cells/dish) were plated in 6-cm dish and then treated with or without CA. After 24 h incubation, the harvested cells were washed with PBS and fixed with 1% glutaraldehyde for 30 min. After washing with PBS, the cells were stained with Hoechst 33258 for 10 min. The cells were washed with PBS, and their nuclear morphology was observed by fluorescent microscopy (Eclipse E600, Nikon, Tokyo, Japan).

### 3.7. DNA Fragmentation Analysis

HL-60 and U937 (1 × 10^6^ cells/dish) were plated in 6-cm dish and then treated with or without CA. After the treatments, the cells were washed with ice-cold PBS and resuspended in lysis buffer (50 mM Tris-HCl, pH 8.0, 10 mM EDTA and 0.5% SDS) with 0.2 mg/mL RNase A for 30 min at 50 °C. Proteinase K was added, and the cells were incubated overnight. The DNA was separated using a 2% agarose gel and visualized under UV illumination after staining with ethidium bromide.

### 3.8. Flow Cytometry Analysis of Apoptotic Cells

Cell cycle analysis to detect the sub-G1 phase cells was performed using a cell cycle phase determination kit (Cayman Chemical, Ann Arbor, MI, USA), according to the manufacturer’s instructions. HL-60 (1 × 10^6^ cells/dish) was plated in 6-cm dish and then treated with or without CA. After treatment with CA for 12 h or 24 h, the cells were centrifuged and washed twice with assay buffer. Then, the cells were fixed with fixative and suspended with staining solution containing propidium iodide (PI) and RNase A. The sub-G1 peak was measured and analyzed in the FL2 channel of a FACSCalibur flow cytometer (Becton Dickinson, San Jose, CA, USA) with a 488 nm excitation laser. The cells (1 × 10^4^ cells) were counted for each sample.

### 3.9. Western Blot Analysis

HL-60 (1 × 10^6^ cells/dish) was plated in 6-cm dish. After treatment with CA for various time periods, the harvested cells were lysed, and the supernatants were boiled for 5 min. The protein concentration was determined using a dye-binding protein assay kit, according to the manufacturer’s manual (Bio-Rad, Richmond, CA, USA). Equal amounts of lysate protein were subjected to SDS-PAGE. The proteins were electrotransferred to PVDF membranes and detected, as described previously [32].

### 3.10. Preparation of Cytosolic and Mitochondrial Fractions

HL-60 (1 × 10^6^ cells/dish) were treated with CA for various periods. To prepare the cytosolic fraction, cells were harvested, washed with PBS and resuspended in ice-cold homogenizing buffer (250 mM sucrose, 20 mM HEPES–KOH (pH 7.5), 10 mM KCl, 1.5 mM MgCl_2_, 1 mM EDTA, 1 mM EGTA, 1 mM DTT, 1 mM PMSF and protease inhibitors). After 30 min incubation on ice, the cell lysates were homogenized for 40 strokes, centrifuged at 100,000*g* for 60 min and the cytosolic fractions were collected. The mitochondrial fraction was isolated using a mitochondria isolation kit, according to the manufacturer’s instructions (Thermo Scientific, Rockford, IL, USA). Equal amounts of cytosolic and mitochondrial proteins were used for the Western blotting.

### 3.11. Flow Cytometry Analysis of the Mitochondrial Membrane Potential (Δψ_m_)

The mitochondrial membrane potential was analyzed using a JC-1 mitochondrial membrane potential detection kit (Minneapolis, MN, USA). Briefly, the cells (1 × 10^6^ cells/dish) were treated with CA for 12 h or 24 h, and a JC-1 staining solution was added to culture medium. After 30 min of incubation at 37 °C, the cells were centrifuged and washed twice with assay buffer. The mitochondrial membrane potential was analyzed using a JC-1 mitochondrial membrane potential detection kit (Minneapolis, MN, USA) with a flow cytometer (FACSCalibur), as described by the manufacturer. The cells (1 × 10^4^ cells) were analyzed at the FL1 channel for green JC-1 monomers and the FL2 channel for red JC-1 J-aggregates with a FACSCalibur flow cytometer (Becton Dickinson, San Jose, CA, USA).

### 3.12. Statistical Analysis

All data were derived from at least three independent experiments. The results were expressed as the mean ± SD in all conditions. Differences between groups were analyzed using the Student’s *t*-test. *p <* 0.05 was considered statistically significant.

## 4. Conclusions

We found that CA was the most potent anti-proliferative triterpene derived from *E. japonica* leaves in leukemia cell lines, but not in normal skin fibroblast cell lines. Analysis of the structure-activity relationships of four triterpenes indicated that the C_2_–C_3_*trans*-dihydroxyl group and the C_19_–C_20_*trans*-dimethyl group are important for suppressing cell proliferation in leukemia cell lines. We showed that CA effectively induced apoptosis in HL-60 cells, which involves the death receptor pathway and the mitochondrial pathway, because CA induced the activation of caspase-8 and -9. Our data also demonstrated that CA-induced caspase-8 activation triggered mitochondrial dysfunction by inducing tBid-mediated Bax activation. These findings suggest that CA might be a potential candidate for the development of anti-cancer drugs for use in the treatment of leukemia, and it would be interesting to determine whether CA-induced apoptosis has an *in vivo* role in anti-cancer activity. We previously reported that crocin [33] and naphthoquinone components from *Alkanna tinctoria* (L.) Tausch [34] exert the anti-cancer effects on human colorectal cancer cells. Further studies are needed to compare the anti-proliferative activity of CA and these compounds.

A number of studies have investigated the anti-cancer potential of triterpenes and their anti-inflammatory, anti-proliferative and pro-apoptotic effects both *in vitro* and *in vivo* [35]. Some of the triterpenes strongly induced apoptosis by altering the mitochondria membrane potential and regulating the expression of Bcl-2 family, survivin proteins and modulating the activation of different caspases [35–37]. Since these bioactive triterpenes seem to act on multiple signaling pathways and cell surface receptors, the *in vivo* and *in vitro* molecular functional analysis of triterpenes are complicated [35–37]. More recently, it has been reported that UA directly bound to CD36, a class B scavenger receptor, in CHO cells stably transected with CD36 [38] and RAW264.7 macrophages [39,40]. Future investigations are needed to compare the binding affinity between CD36 and four triterpenes of *E. japonica* leaves for deeply understanding the structure–activity correlations.
